# A Case of Muir-Torre Syndrome

**DOI:** 10.7759/cureus.14582

**Published:** 2021-04-20

**Authors:** Radhika Sheth, Priya Menon, Devin Malik

**Affiliations:** 1 Internal Medicine, Henry Ford Health System, Jackson, USA; 2 Hematology/Oncology, Henry Ford Health System, Jackson, USA

**Keywords:** muir-torre syndrome, hnpcc, lynch syndrome, mismatch repair genes, colon cancer

## Abstract

Muir-Torre syndrome (MTS) is an autosomal dominant condition characterized by dermatological tumors along with visceral malignancies. The dermatological manifestations include recurrent sebaceous adenomas and keratoacanthomas. The commonly seen visceral malignancies are colorectal, gynecological, and urological. It is a variant of hereditary non-polyposis colorectal carcinoma syndrome (HNPCC). The underlying genetic mechanism is germline mutations in the DNA mismatch repair (MMR) genes leading to microsatellite instability (MSI), conferring an increased risk of developing malignancies. This is a case of a 57-year-old male patient with a history of colon cancer diagnosed at age 32 and multiple sebaceous adenomas. The patient also has a strong family history of cancer. They were referred to oncology after the immunohistochemical staining of a sebaceous adenoma showed loss of expression for MSH2 and MSH6. Next-generation sequencing identified a mutation in the MSH2 gene. These patients require genetic testing, counseling, and close follow-up with regular screening for cancer.

## Introduction

Muir-Torre syndrome (MTS) was first described independently by Muir [1] in 1967 and Torre [2] in 1978. It is an uncommon disease that is clinically defined by the presence of one or more sebaceous gland malignancy in conjunction with at least one internal malignancy. It is believed to be a phenotypic variant of Lynch or hereditary nonpolyposis colorectal carcinoma (HNPCC) syndrome because they are both associated with similar molecular pathogenic mechanisms. MTS and HNPCC syndrome arise due to mutations in the DNA mismatch repair (MMR) genes that lead to the accumulation of errors in the genome and eventual development of cancer [3,4]. About two-thirds of MTS cases are not associated with MMR defects and they are included under MTS II [5]. The pattern of inheritance is autosomal dominant but cases of MTS II are more often sporadic [6].

The most commonly observed cutaneous malignancies in these patients are sebaceous adenomas and keratoacanthomas [7]. Colorectal carcinomas are by far the commonest visceral malignancies associated with MTS. Testing of the tumors with immunohistochemistry (IHC) to look for loss of MMR gene expression and microsatellite stability analysis [4,8], along with gene sequencing, is central to the diagnosis. Patients with even a single sebaceous malignancy should be prompted for a thorough history of cancer and undergo testing with IHC for screening [9,10]. Patients must be referred to genetic counseling to identify at-risk family members. MTS requires an interdisciplinary effort from the physician’s end as these patients need to be very closely monitored for the emergence of cancers.

## Case presentation

**Figure 1:**
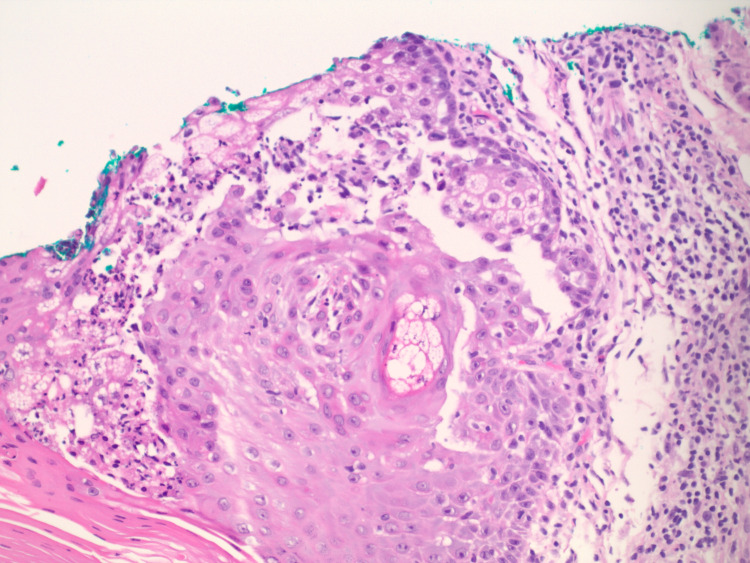
Hematoxylin and eosin-stained section of the left upper cheek showing a sebaceous adenoma.

**Figure 2:**
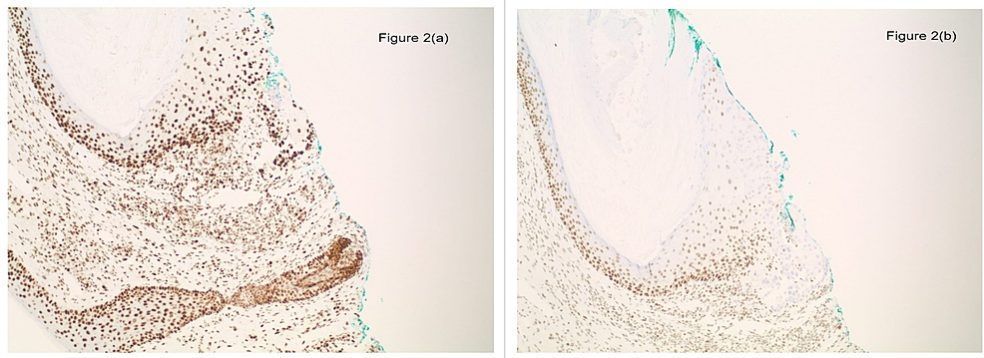
Immunohistochemistry staining of the sebaceous adenoma demonstrating retained expression of PMS2 in 2(a) and loss of MSH6 in the section in (b).

We describe here a case of a 57-year-old male patient who was referred to the oncology office from the dermatologist’s office. The dermatologist had performed a shave biopsy of a papule on his left cheek and the biopsy showed a sebaceous adenoma (Figure 1. Further immunohistochemical staining demonstrated a loss of expression for MSH2 and MSH6 and retained expression for MLH1 and PMS2 within the sebaceous neoplasm (Figure 2). The patient has a past medical history of colon cancer diagnosed at age 32, treated with chemotherapy and a partial right hemicolectomy with ileocolic anastomosis, squamous cell carcinoma of the right clavicle treated with excision, multiple benign rectal polyps, sebaceous cysts, and adenomas. He also had a family history of malignancies including breast and lung cancers in his mother, renal cancer in his father, and other unknown malignancies in second-degree relatives. He smoked a pack of cigarettes a day for 44 years. The physical examination was unremarkable.

With the strong family history, an early diagnosis of colon cancer, and now a sebaceous adenoma showing loss of MSH2 and MSH 6 expression, we suspected that the patient had Muir-Torre syndrome (MTS). He underwent next-generation sequencing which showed a deletion of MSH-2 del promoter exon 6 and confirmed the diagnosis of MTS. The genetic results further identified the patient as being at high risk for colon and gastric cancers and at an elevated risk of developing pancreatic and/or prostate malignancies.

He was referred to a geneticist for genetic counseling. He was informed about his family members’ and his increased risk for developing cancers, and the importance of regular follow-up and surveillance. No further genetic testing was recommended by the geneticist. We had discussions with dermatology, gastroenterology, and urology. Our current surveillance plan is for the patient to undergo annual skin checks, colonoscopy every 1-2 years, and upper endoscopy every 3-5 years. Due to a history of renal cancer in a first-degree relative, loss of MSH2 expression, and the relative inexpensiveness of the studies, annual screening of urological malignancies with urinalysis and prostate-specific antigen were also recommended to the patient.

## Discussion

Muir-Torre syndrome (MTS) is an exceedingly rare autosomal dominant condition characterized by at least one cutaneous sebaceous malignancy and at least one visceral malignancy. Very little is known about the exact incidence and distribution, but it shows a slight male predominance with a male to female ratio of 3:2. It is believed to be a clinical variant of hereditary nonpolyposis colorectal cancer (HNPCC) or Lynch syndrome because of similarities in clinical features and molecular mechanisms. They both arise in the setting of mutations in the DNA mismatch repair (MMR) genes [11,12].

The MMR gene family includes MLH1, MSH1, MSH2, MSH6, and PMS2. Together these genes are responsible for maintaining genomic integrity and repairing base mismatches during DNA replication. Inactivation of or mutation in the MMR genes leads to the accumulation of replication errors, particularly in microsatellite regions. Microsatellites are short repetitive segments of bases in the DNA which are particularly sensitive to base mismatch errors. The molecular pathogenesis of MTS involves germline mutations in MMR genes, most commonly the MSH2 gene, leading to microsatellite instability (MSI) [8,13]. Virtually all tumors seen in MTS have a high-frequency MSI (MSI-H) phenotype. Unlike HNPCC syndrome, which may involve any MMR gene, MTS is more commonly associated with a mutation in the MSH2 gene [14]. About 35% of MTS cases are not associated with MSH2 mutation but instead are due to biallelic inactivation of the base excision repair gene MHY. They are considered to be MTS type II, a variant where tumors do not display MSI. In contrast to MTS I, which shows germline mutations in MMR genes and is associated with early-onset colorectal carcinomas, MTS II inheritance is autosomal recessive and the tumors are sporadic and tend to develop later in life [5,7].

By far, the most commonly seen sebaceous gland tumors in MTS are sebaceous adenomas and keratoacanthomas [6]. Sebaceous adenomas are fleshy, yellow papules or nodules with or without ulceration or umbilication. They most commonly involve the trunk but can also be seen on the face, especially around the eyes. Keratoacanthomas are dome-shaped nodules with keratin-filled centers usually seen on sun-exposed areas. The presence of ectopic sebaceous glands, called Fordyce granules, can also be seen in MTS. They manifest as yellow macular or papular lesions on the buccal mucosa. Some patients may also develop sebaceomas and basal cell carcinoma with sebaceous differentiation [7]. These cutaneous manifestations precede the appearance of internal malignancies [15]. Colorectal cancer is the most frequently associated visceral cancer in MTS, followed by gynecological and urological cancers. Not infrequently, patients with MTS may also develop cancer involving the breast and the upper gastrointestinal tract. Colorectal cancers in MTS involve the proximal colon and the median age of diagnosis is 50 years [6,16]. 

**Figure 3:**
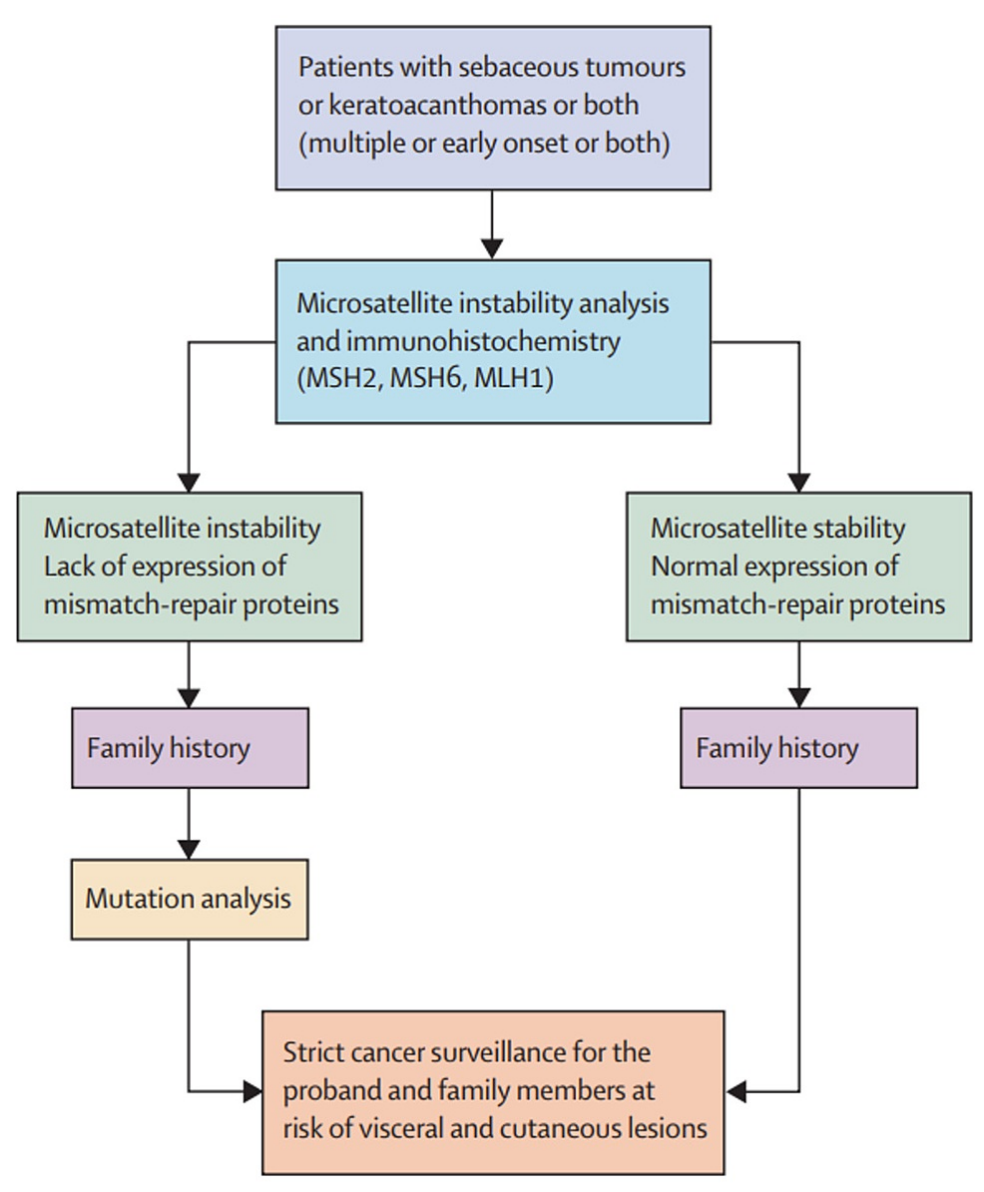
Proposed algorithm for the diagnosis of MTS. MTS: Muir-Torre syndrome Source: Ponti and Ponz de Leon [6], reprinted with permission

The diagnostic criteria of MTS include the presence of at least one sebaceous and at least one internal malignancy. In a study of 90 patients with sebaceous neoplasms (SN), Roberts et al. proposed a scoring system to determine the risk of MTS in these patients by taking into account the age of diagnosis, number of sebaceous neoplasms, family history, and personal history of Lynch syndrome [17]. In a retrospective study of 86 patients with SN referred for genetic evaluation, 25% were found to have germline MMR mutations, and out of 77 patients who underwent immunohistochemistry (IHC), 38 had defects in MMR proteins [9]. A study in Denmark looked at the prevalence of MTS in patients with SN and found that 18 cases out of the total 32 showed deficient MMR proteins on IHC [10]. Based on these studies and the rarity of sebaceous neoplasms, it has been recommended that all sebaceous neoplasms be tested by IHC as a screening test for MTS. Any patient presenting with sebaceous neoplasm warrants asking for a detailed personal and family history of cancer. MSI analysis should be done for those that have loss of MMR proteins on IHC. If the tumor shows abnormal IHC and presence of MSI, genomic sequencing should be performed to identify germline mutations in MSH2, MSH6, MLH1, or PMS2. MTS II tumors show microsatellite stability and do not show MMR protein defects on IHC. MYH gene analysis should be considered in these cases. Figure 3 provides a proposed algorithm for identifying MTS.

Most cutaneous tumors in MTS can be treated with cryotherapy or excision. Sebaceous carcinoma involving the ocular area can be aggressive and metastasize. They require wide local excision either with Mohs micrographic surgery or standard technique. These tumors usually do not respond well to radiation and it should be used as an adjuvant treatment to excision, or in palliative cases and for those unable to tolerate surgery. There is also a higher rate of recurrence with radiation treatment alone [7]. As such there are no established treatment guidelines, but there have been reports of cases where low-dose isotretinoin and interferon-alpha [18] have been tried for prophylaxis with limited success.

Similar to HNPCC syndrome, patients with MTS and high-risk family members should be referred to genetic counseling and undergo regular surveillance for cancer [6]. They need strict follow up and this requires a multidisciplinary effort. The patient should have a dermatological examination annually to look for suspicious lesions. They must also have annual physical exams including breast and pelvic exams in women, and testicular exams in men. They should undergo screening colonoscopies every three to five years starting at age 25 or five years before the youngest age of diagnosis of colorectal carcinoma in a family member. Regular screening with transvaginal ultrasounds and endometrial biopsies should be done to look for gynecological malignancies. In families with a history of gastric cancer, upper endoscopies can be considered. Table 1 lists the suggested screening tests and their frequency in these patients based on proposals by Ponti and Ponz de Leon [6] and Cohen et al. [16]. 

**Table 1:** Suggested screening tests and screening intervals.

Test	Timing
Colonoscopy	Every 3-5 years, starting at an age of 25-30 (or earlier if family history of colon cancer diagnosis at an age earlier than 25 years)
Upper gastrointestinal (GI) endoscopy	Every 3-5 years, starting at an age of 30-35 years (if there is a positive family history of upper GI cancers)
Mammogram	Every 1-2 years in women >50 years of age
Pap smear	Every 3-5 years in women, starting from an age of 21 years
Endometrial biopsy	Every 3-5 years in women, starting at an age of 50 years
Urinalysis	Annually
Tumor markers- including carcinoembryonic antigen (CEA) and prostate-specific antigen (PSA)	Annually, based on risk factors
Physical examination, including a dermatological exam	Annually, including at presentation

In conclusion, Muir-Torre syndrome is a rare condition that predisposes the patient to developing multiple cutaneous and visceral malignancies early in life. Long-term follow-up and close surveillance are vital in these patients- which has important implications in primary care and oncology.

## Conclusions

Muir-Torre syndrome is a rare autosomal dominant condition, related to Lynch syndrome, and is associated with mutations in the DNA mismatch repair (MMR) genes, particularly MSH2. The diagnostic criteria include the presence of at least one sebaceous malignancy with at least one visceral malignancy. For patients presenting with sebaceous cancer, immunohistochemical staining of the tumor for MMR proteins is recommended, and then testing for microsatellite instability. Identification of the mutation through next-generation sequencing is now considered the standard. Genetic counseling for risk identification and close follow up for cancer screening is especially important in these cases.
